# Anxiety-like features and spatial memory problems as a consequence of hippocampal SV2A expression

**DOI:** 10.1371/journal.pone.0217882

**Published:** 2019-06-05

**Authors:** Maria Elisa Serrano, Odile Bartholomé, Priscilla Van den Ackerveken, André Ferrara, Bernard Rogister, Alain Plenevaux, Ezio Tirelli

**Affiliations:** 1 GIGA-CRC In vivo imaging, University of Liège, Liège, Belgium; 2 GIGA-Neurosciences, University of Liège, Liège, Belgium; 3 Department of Psychology, University of Liège, Liège, Belgium; 4 Neurology Department, CHU, Academic Hospital, University of Liège, Liège, Belgium; University of Modena and Reggio Emilia, ITALY

## Abstract

The Synaptic Vesicle Protein 2A (SV2A) is a transmembrane protein whose presence is reduced both in animal models and in patients with chronic epilepsy. Besides its implication in the epileptic process, the behavioural consequences of the changes in its expression remain unclear. The purpose of our research is to better understand the possible role(s) of this protein through the phenotype of cKO (*Grik4 Cre+/-*, *SV2A lox/lox*) mice, male and female, which present a specific decrease of SV2A expression levels in the hippocampal glutamatergic neurons but without any epileptic seizures. In this study, we compare the cKO mice with cHZ (*Grik4 Cre+/-*, *SV2A lox/+*) and WT (*Grik4 Cre+/+*, *SV2A lox/lox*) mice through a battery of tests, used to evaluate different features: the anxiety-related features (Elevated Plus Maze), the locomotor activity (Activity Chambers), the contextual fear-related memory (Contextual Fear Conditioning), and the spatial memory (Barnes Maze). Our results showed statistically significant differences in the habituation to a new environment, an increase in the anxiety levels and spatial memory deficit in the cHZ and cKO groups, compared to the WT group. No statistically significant differences due to the genotype appeared in the spontaneous locomotor activity or the fear-linked memory. However, sexual differences were observed in this last feature. These results highlight not only an important role of the SV2A protein in the cognitive and anxiety problems typically encountered in epileptic patients, but also a possible role in the symptomatology of other neurodegenerative diseases, such as the Alzheimer’s disease.

## Introduction

The SV2 protein family comprises three integral membrane paralogs: SV2A, SV2B, and SV2C. In spite of sharing approximately 60% of their sequences, these three isoforms are implicated in different pathologies, thus suggesting a specific role for each of them [1–4]. Amongst these isoforms, the most studied is the SV2A protein, due to its ubiquitous expression in the brain and its implication in epileptic disease (see hereunder). Beyond the initial suggestion of its role as a transporter of ions or neurotransmitters [5,6], the SV2A protein seems to act mainly as modulator of the synaptic transmission. The invalidation of this protein does not alter the morphology of the brain, the amount of synapses, or their structure [6,7]. However, its absence reduces the neurotransmission [8,9] and may induce an imbalance between glutamatergic and GABAergic levels [10–13]. Furthermore, three key findings have identified the SV2A protein as an all-important molecule involved in the epileptic process. Accordingly, the authors in [7,14] showed that homozygous SV2A KO mice displayed severe seizures at P7, and died in *status epilepticus* two to three weeks after birth. In humans, the homozygous mutation in SV2A has been also associated with intractable epilepsy, microcephaly, and developmental and growth retardation [15]. Moreover, SV2A has been proved to be the specific molecular target of new antiepileptic drugs, such as the levetiracetam [16] or the brivaracetam [17,18].

Different studies have explored the role of the SV2A protein in epilepsy: in humans, several epileptic disorders are related with a reduction in the amount of SV2A [1,19,20], suggesting a role of this protein in the epileptogenesis and/or the ictiogenesis [12,13,21]. However, the possible implication of SV2A in the cognitive impairment and mood disorders accompanying epileptic disease is still unknown. In light of this, several articles have associated the use of levetiracetam with memory and executive function enhancement [22–24]. As for other pathologies, the use of this antiepileptic drug has highlighted a pro-cognitive effect as well [25–28], suggesting a role of SV2A in cognition.

The studies carried out with male knockout mice have also provided interesting insights regarding the role of the SV2A protein in cognition. In particular, the phenotyping of the male heterozygous SV2A (+/-) mice revealed no motor differences but rather anxiety-like features in these mice compared with the wild type [29]. Recently, a cKO mouse model (*Grik4 Cre+/-*, *SV2A lox/lox*) has been produced and validated by our laboratory using the Cre/loxP recombination system [30]. This model is characterized by a decrease of the expression of the SV2A protein specifically in the glutamatergic pyramidal neurons of the hippocampus, one of the most affected brain regions in epilepsy [31–34]. Furthermore, this cKO model, in contrast to the SV2A homozygous or heterozygous mice, does not present an epileptic or pro-epileptic phenotype (characterized by a lower epileptic threshold, evaluated with the *pentylenetetrazol* test) [30], thus increasing their survival and facilitating their evaluation in different tests [7,14,35].

In this article we evaluate the consequences of the hippocampal downregulation of the SV2A protein in the phenotype of *Grik4 Cre+/-*, *SV2A lox/lox* mice. Using both, males and females, we studied the possible existence of sex-differences in the cognitive and behavioural processes evaluated, some of them already reported in epileptic patients [36–38]. Thus, the main goal of this research is to unravel the possible role of this protein in the cognitive and behavioural processes associated with the hippocampal structure, offering useful insights into the role of SV2A, not only in the pathology but also in the healthy brain.

## Materials and methods

### Animals

Homozygous SV2A conditional knockout mice of both sexes (cKO; *Grik4 Cre+/-*, *SV2A lox/lox*), presenting a decrease of SV2A protein expression in excitatory glutamatergic neurons of the dentate gyrus and the CA3 hippocampal area, were generated by using the Cre/loxP recombination system, following the procedure published in [30]. Their phenotype was compared with such of the heterozygous (cHZ; *Grik4 Cre+/-*, *SV2A lox/+*) and with the phenotype of wild type mice (WT; *Grik4 Cre+/+*, *SV2A lox/lox*). All animals were genotyped by PCR following the protocol described in [30].

A total of 141 mice were employed: 66 males and 69 females. Amongst these animals, 27 males and 36 females were also evaluated using the Barnes Maze test to detect spatial learning or memory problems specifically associated with the hippocampus.

Note that, in principle, for a power of 0.8 and at a critical threshold of 0.05 (alpha), these sample sizes are able to detect medium to very large effects (η_p_^2^ > 0.14), but not small effects which require greater sample sizes [39]. Related calculations were carried out with G*Power software [40].

### Experimental design

Two weeks before starting the experiments, six-week old mice belonging to the three genotypes were housed in individual standard transparent poly-carbonate cages (31.5 cm (L) × 15.5 cm (W) × 13 cm (H)) with pine sawdust bedding. During all the experimental procedure, the distance between the cages allowed the animals to maintain visual, olfactory and acoustic interactions. Furthermore, tap water and food (standard pellets, Carfil Quality, Oud-Turnhout, Belgium) were provided *ad libitum*. The animal room was maintained on a 12:12h dark-light cycle (lights on at 7:30 am, off at 7:30 pm), at an ambient temperature of 20–24°C.

**Fig 1:**
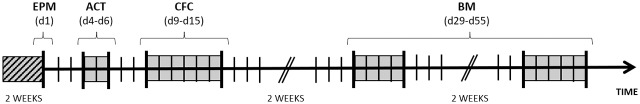
Experimental time-line and design. After two weeks of accommodation to the animal laboratory facilities, 69 males and 72 females belonging to the three genotypes (WT, cHZ, and cKO) were evaluated for anxiety-related behaviour (EPM, d1), spontaneous locomotor activity (ACT, from d4 to d6), and contextual fear conditioning memory (CFC, from d9 to d15). Following two weeks of recovery, the spatial memory was assessed (BM, from d29 to d55) with a procedure divided in four parts with a duration of 4 + 1 days, a period of consolidation of two weeks, and 4 + 2 days respectively. Abbreviations: WT: *Grik4 Cre+/+*, *SV2A lox/lox*; cHZ: *Grik4 Cre+/-*, *SV2A lox/+*; cKO: *Grik4 Cre+/-*, *SV2A lox/lox*; EPM: Elevated Plus Maze; ACT: Activity chambers; CFC: Contextual fear conditioning; BM: Barnes Maze.

When the mice were eight weeks old, the experimental procedure started, performing the tests during the 12h light cycle (between 9 a.m. and 3 p.m.) (see Fig 1). The first day, the anxiety-related behaviour was evaluated with the Elevated Plus Maze (EPM). From days four to six, the spontaneous locomotor activity and the habituation to the environment was evaluated with activity chambers (ACT). The contextual fear conditioning memory was assessed with the contextual fear conditioning test (CFC), conducted as follows: on day nine (acquisition trial), mice were placed in the conditioning chamber for the context (conditioned stimulus, CS)–shock (unconditioned stimulus, US) pairing; then, the contextual fear conditioning memory was evaluated one hour, one day, and six days later. Finally, the spatial memory was assessed two weeks later with the Barnes Maze test (BM) using the following method: (1) the mice were trained during four consecutive days (acquisition trials) to spatially locate a target hole in the Barnes Maze; (2) the spatial memory of the mice was evaluated the following day (probe trial); (3) after two weeks, mice were subject to the same 4-day training in order to assess their memory retention and to determine which level of performance they were able to reach; (4) the first two days after this last training, the cognitive flexibility of mice was tested in a reversal learning procedure. At the end of the experimental procedure (second day of reversal learning), animals were sacrificed by cervical dislocation.

The three parts of the experimental process (testing, scoring, and statistical analysis) were blinded: animals were identified as members of A, B, or C groups, without information about the genotype associated to the letters until the generation of results. This course of action eliminates intentional or subconscious bias which could interfere in the experimental procedure.

The experimental procedures and protocols used in this investigation were reviewed and approved by the Institutional Animal Care and Use Committee of the University of Liege (dossiers 1258 and 1573), according to the Helsinki declaration, and conducted in accordance with the European guidelines for care of laboratory animals (2010/63/EU). All efforts were made to minimise the number of animals used and their suffering. Moreover, the ARRIVE guidelines (Animal Research Reporting In Vivo Experiments) [41] was followed as closely as possible to confer a minimal intrinsic quality to the study.

### Anxiety-related behaviour: EPM

The EPM was used to evaluate fearfulness/anxiety in mice in a single session. This apparatus consists of two open and two closed arms (29 cm (L) × 5 cm (W) × 2.5 cm (H) each one) emerging from a central platform (5 cm × 5 cm) to form a plus shape, which sits 80 cm above the ground level. The floor and walls of the enclosed arms were made of black hard plastic (Forex), while the floor of the open arms was made of grey hard plastic (Forex). The behaviour of the mice was recorded with a webcam (Logitech QuickCam Pro 5000).

For testing, each mouse was placed in the central platform, facing the left open arm, and was allowed to freely explore the maze during five minutes [42]. After each trial the floor was wiped clean with Ethanol 70% and dried.

Two parameters were evaluated: (1) the percentage of entries in the open arms with respect to the total (open / open + closed), and (2) the percentage of time spent in the open arms with respect to the total testing time (5 minutes). An entry was scored when the mouse had entered the arm with all four paws. The natural aversion of rodents for open spaces along with the relationship between anxiogenic drugs and a reduction in the percentage of time spent in the open arms, make both parameters reliable measures of anxiety in mice [43–45]. Data (see S1 Data) were analysed with a two-way 3 × 2 ANOVA, considering the *Genotype* (3 levels) and the *Sex* (2 levels) as between-group factors.

### Locomotor activity: ACT

The spontaneous locomotor activity and the capacity of habituation to an environment were evaluated in individual activity chambers (Columbus Instrument, Ohio, USA), for three consecutive sessions (one per day). Each chamber consisted in an enclosure with PVC opaque black walls and a smooth floor (20.30 cm (L) × 20.30 cm (W) × 20.30 cm (H)) with eight infrared light-beam sensors 1.54 cm above the chamber floor and spaced 2.54 cm apart. The interruption of two consecutive sensors in a chamber was detected by a central computer and measured as a locomotion count by the recording software (Opto-Max Activity Meter, Ohio, USA).

Mice were placed at the middle of the chamber and allowed to freely explore during one hour [42,46]. After each individual session, the floor and the walls of the chambers were cleaned with Ethanol 70% and dried.

The estimation of the travelled distance (in cm) was provided by the recording software for each 60-minute session. Data (see S2 Data) were assessed with a mixed-model 3 × 2 × 3 ANOVA, incorporating the *Genotype* and the *Sex* as between-group factors and the repeated exposure to the apparatus (*Session*) as a within-subject factor (3 levels).

### Contextual fear conditioning memory: CFC

The contextual fear conditioning memory was longitudinally assessed at three different time points to detect possible group differences in memory retention. The apparatus used, an automated rodent conditioning system (MED Associates Inc., St. Albans, VT, USA, ENV-307W-TH), consists of two identical conditioning chambers (24 cm (L) × 20 cm (W) × 21.5 cm (H)) each enclosed in a sound-attenuating cubicle with walls and ceiling constructed of clear Plexiglas. The floor of each chamber consisted of 23 stainless steel rods (3 mm in diameter, 8 mm apart). The chambers were illuminated by a standard single house light, mounted at the top centre of the right wall. A software program controlled a shock scrambler that delivered the footshock (US) through the floor rods. Stimuli presentation and data recording from both boxes were controlled by a MED-PC program via a specific interface.

The procedure followed was inspired by Kimura’s work [47], with a training session and several sessions to evaluate the CFC memory:

#### Acquisition trial

The mice were placed in the test chamber (CS), previously cleaned with 70% Ethanol. A lighted up lightbulb indicated the starting of every session, consisting of a pre-shock period of 5 minutes (basal measure) followed by three moderate footshocks (US) of 2 seconds and an intensity of 0.5 mA, administered every 60 seconds. After the last footshock, the mice remained in the chamber for another 60 seconds.

#### CFC evaluation

The mice were exposed to the CS in three different sessions: 1h, 24h and 6 days after the acquisition trial. The exposition consisted in a 5-minute session without any footshock being delivered.

In all the sessions the parameter analysed was the percentage of freezing time, defining freezing as the total absence of movements (except those related to breathing). This behaviour is considered as part of a defensive mechanism relevant to perception and action preparation in presence of stimulus or situations perceived to be threatening. In humans, there is evidence that the freezing reaction is also present [48,49]. Data (see S3 Data) were analysed with two mixed-model 3 × 2 × 4 ANOVA. The first one served us to analyse the acquisition trial, incorporating the *Genotype* and the *Sex* as between-group factors, and the time pre- (5 minutes) and post- (1 minute) footshock as a within-subject factor (*NumFootshock*, 4 levels). With the second one we analysed the percentage of freezing during the CFC evaluation, using the same between-group factors than during the acquisition, and the time pre- (5 minutes) and post- (1 minute) footshock as a within-subject factor (*NumFootshock*: 4 levels).

### Spatial memory: BM

We employed the BM (Med Associates, Inc., United Kingdom) to study the spatial memory of mice. The apparatus consisted of a white, circular table of 122 cm in diameter, elevated 140 cm above the ground floor, with 18 equally spaced holes of 5 cm in diameter. The aversive stimulation was provided by four LED lights surrounding the maze to produce high-intensity lighting in the centre of the maze of 1000 lux. A black acrylic box (escape box) (15 cm (L) × 12.5 cm (W) × 40 cm (H)) was placed under one of the 18 holes (different for each mouse) in order to provide the mice with protection against the light. A metallic ramp facilitated its access. Three black spatial cues (circle, triangle, and cross) with dimensions 210 mm (L) x 297 mm (H), were fixed to the room walls at the BM level (in height), and at 25 cm of the apparatus. A webcam (Logitech QuickCam Pro 5000) connected to a computer was located over the maze to record the mouse performance.

For each trial, mice were deposited into a black box (7 cm (L) x 6 cm (W) x 5 cm (H)) covered with a black cloth and laid in the centre of the maze for 30 seconds. The box was then lifted, and the mouse was allowed to explore the area to find the escape hole. The trial ended when the mouse completely entered on the metallic ramp (the four paws on the ramp). If the mouse did not enter the escape box within 3 min, it was gently conduced to the target hole. Once inside the box, the entry was shut and the mouse was kept inside during 1 min before it was returned to its home cage. The apparatus was cleaned with 70% ethanol after each trial.

The trials were grouped in four phases (acquisition, probe, long-term acquisition, and reversal learning), following a protocol inspired by Kesby’s work [50,51] and Carrillo-Mora’s advices about spatial memory evaluation [52] (see S4 Data):

#### Acquisition trials

Sixteen acquisition trials were performed on four consecutive days (days 1 to 4), with 4 × 3-minute trials per day and an inter-trial time ranging from 15 to 20 minutes. Data were analysed using a mixed-model 3 × 2 × 4 ANOVA, considering the *Genotype* and the *Sex* as between-group factors and the *Days* as a within-subject factor (4 levels).

#### Probe trial

For the one-day probe trial (day 5), used to assess the spatial memory of mice, the escape box was removed and the spatial memory of mice was tested during 90 seconds. Data were analysed with a two-way 3 × 2 ANOVA, with the *Genotype* and the *Sex* as between-group factors. We also employed the nonparametric Chi-Square Test of Association to determinate if there was a relationship between the *Genotype* and the *Strategy* (3 levels) employed to reach the target.

#### Long-term acquisition trials

Two weeks after the probe test, mice followed an identical protocol to the acquisition trials (days 19 to 22) to assess the capacity of mice to recover the spatial learning acquired and to determine which level of performance they were able to reach. Data were analysed using a mixed-model 3 × 4 ANOVA, considering the *Genotype* as a between-group factor (3 levels) and the *Days* as a within-subject factor (4 levels).

Additionally, the first day (day 19) was considered as a measure of the *long-term retention* [53,54], assessed with a two-way 3 × 2 ANOVA, with the *Genotype* and the *Sex* as between-group factors. The escape box was available during all these trials.

#### Reversal learning

The two consecutive days after the long-term acquisition trials (days 23 and 24), the cognitive flexibility of mice was tested with the reversal learning procedure. Each day consisted of 4 x 3-min trials identical to the acquisition trials, positioning the escape box on the opposite side of the table. Data from the last day of long-term acquisition (day 25) and the two days of reversal learning were analysed with a mixed-model 3 × 2 × 3 ANOVA, considering the *Genotype* and the *Sex* as between-group factors and the *Days* as a within-subject factor (3 levels).

Three parameters were measured in all the trials: (1) the latency (time in seconds to enter the target box), (2) the number of errors (nose pokes and head deflections over any hole that did not have the target box), and (3) the percentage of use of the spatial strategy (the mouse only visited the escape hole and/or the two next ones).

During the probe trial we also evaluated the time spent by each mouse in the quadrant of the maze that contained the target hole, in the centre, and in the rest of the maze. Furthermore, for each group, we evaluated the preference for the use of three different strategies to find the target hole: the spatial strategy, the serial strategy (systematic search taking it to consecutive holes or to every second hole), and the random strategy (the mouse undertakes an unorganized search or searches of separate holes crossing the maze centre).

### Data analysis

The statistical analyses were carried out with SPSS (IBM SPSS Statistics 25; USA). GraphPad Prism 5 (GraphPad Software, Inc.; USA) was used to graphically represent the results.

For the experiments, the Levene’s test was used to evaluate the assumption of homogeneity of variance. In the experiments including within-subject measures and following a significant Mauchly’s test, the Greenhouse–Geisser correction (G.G.) was used to adjust for potential violations of the assumptions of compound symmetry and sphericity. As we have previously detailed in the sections pertaining to each of the tests, data were mainly assessed with two-way ANOVA (EPM; probe trial and long-term retention of BM), mixed-model ANOVA (ACT; CFC; reversal learning, acquisition and long-term acquisition trials of BM), and the chi-square test of association (probe trial of BM). The meaningful between-mean differences were assessed via Tukey’s HSD test (post-hoc) or Bonferroni test derived from the appropriate mean-square error-term. The critical threshold of statistical significance was always p < .05. Partial eta squared (η_p_^2^) are reported as a measure of effect size [39].

## Results

### Anxiety-related behaviour: EPM

Regarding the percentage of entries, there were no statistically significant differences due to the *Sex* (p > .117). However, there was a main effect of the *Genotype* (η_p_^2^ = 0.085; F_2,129_ = 5.99, p = .003), and a significant effect of the interaction *Sex* × *Genotype* (η_p_^2^ = 0.047; F_2,129_ = 3.18, p = .045). Further pairwise comparisons brought out significant differences between WT and cHZ groups (p = .007), and WT and cKO groups (p = .014). When the effect of the interaction *Sex* × *Genotype* was analysed in depth, the results highlighted that these differences were significant only in the group of males (WT vs cHZ: p = .032; WT vs cKO: p = .001), but not in the group of females (p > .265).

**Fig 2:**
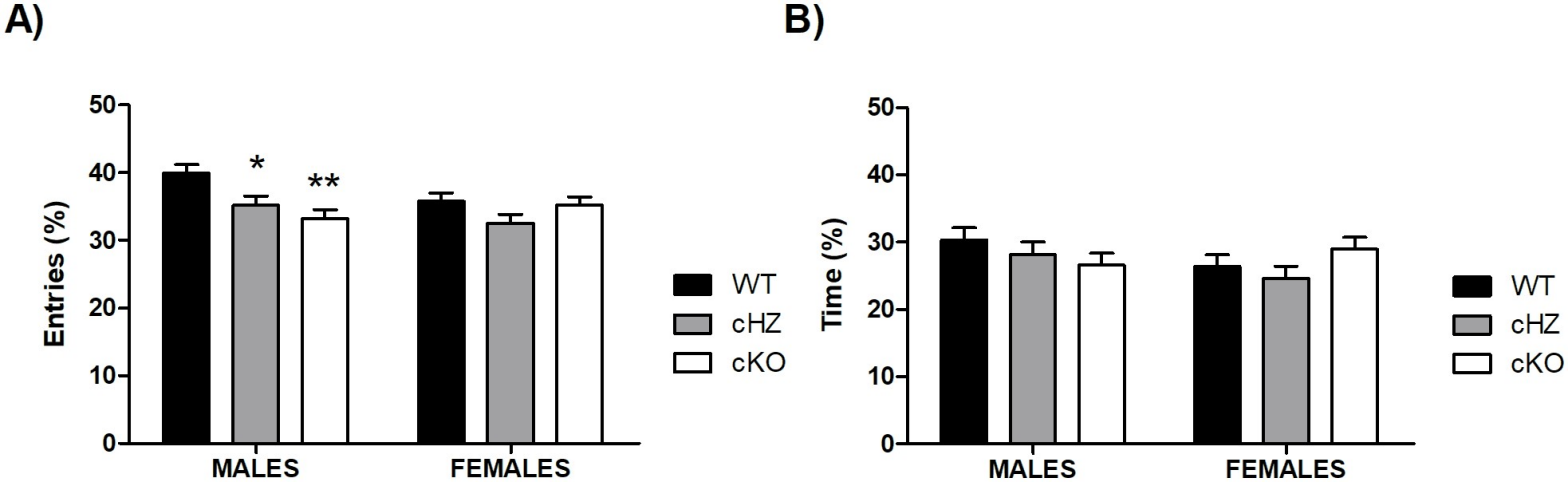
Anxiety-related behaviour: EPM. The bar plots represent the Mean and the SEM of the percentage of entries (**A**) and the percentage of time (**B**) in the open arms during the 5-minute trial. Three groups were compared: WT (*Grik4 Cre+/+*, *SV2A lox/lox*, n = 45), cHZ (*Grik4 Cre+/-*, *SV2A lox/+*, n = 47), and cKO (*Grik4 Cre+/-*, *SV2A lox/lox*, n = 43), with Males (n = 22) and females (n = 23). (*) and (**) indicates that both, cHZ and cKO groups are statistically significant to the WT group, as yielded by a Tukey HSD test taken at p < .05 and p < .01, respectively.

Concerning the percentage of time spent in the open arms, no statistically significant differences were found due to the variables *Sex*, the *Genotype*, or the interaction between both (all p > .145) (see Fig 2).

### Locomotor activity: ACT

The statistical analyses did not reveal any significant differences due to the *Genotype*, the *Sex*, or the interaction *Genotype × Sex* in the distance travelled, with all p > .598.

**Fig 3:**
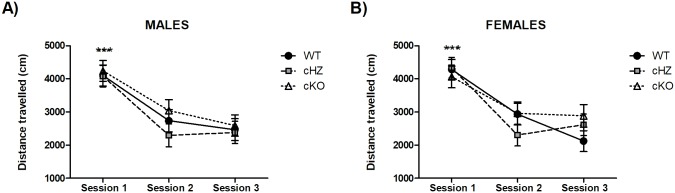
Locomotor activity: ACT. The lines represent the distance in centimetres travelled by the mice during the 60-minute session (Mean and SEM). The measure was acquired in three consecutive sessions, comparing the groups WT (*Grik4 Cre+/+*, *SV2A lox/lox*), cHZ (*Grik4 Cre+/-*, *SV2A lox/+*), and cKO (*Grik4 Cre+/-*, *SV2A lox/lox*). Males (n = 22) are presented in subfigure **A**, whereas females (n = 23) are shown in subfigure **B**. (***) indicates that the corresponding marginal mean (main effect of *Session*) is significantly different from the other sessions, as yielded by a Bonferroni test taken at p < .001. Note that in cHZ and cKO groups no statistically significant differences were found between the second and the third sessions (all p > .395).

However, the variable *Session* (η_p_^2^ = 0.509; F_2,256_ = 132.75, p < .001), and the interaction *Genotype × Session* (η_p_^2^ = 0.047; F_4,256_ = 3.13, p = .016) were significant. Further pairwise comparisons, using the Bonferroni procedure, highlighted differences between the first session and the rest (p < .001) in all the groups. When we analysed the effect of the interaction *Genotype × Session*, we could observe that the second and the third sessions were only significant in the WT group, but not in the cHZ (p > .763) or the cKO group (p > .395). This phenomenon is more clear in the females than in the males. Nevertheless, the interactions *Sex × Session* or *Genotype × Sex × Session* were not significant (all p > .320). Results are represented in Fig 3.

### Contextual fear conditioning memory: CFC

#### Acquisition trial

During the acquisition trial, no statistically significant differences were found due to the variables *Genotype*, the interaction *Genotype × NumFootshock* or the interaction *Genotype × Sex* (all p > .442).

Nevertheless, there was a main effect of the *Sex* (η_p_^2^ = 0.062; F_1,130_ = 8.56, p = .004), a significant effect of the repeated administration of footshocks (η_p_^2^ = 0.526; F_2,242_ = 144.12, p < .001), and a significant effect of the interaction *Sex × NumFootshock* (η_p_^2^ = 0.60; F_2,242_ = 8.36, p < .001). Bonferroni’s pairwise comparisons evidenced statistically significant differences in the percentage of freezing through the acquisition trial, with a significant increase after each footshock (p < .001). Furthermore, significant differences were found between males and females after the second and the third footshock, with a higher increase in the time of freezing in the females compared to the males.

There were no statistically significant differences due to the interaction *Genotype × Sex × NumFootshock*, with p = .95.

#### CFC evaluation

During the CFC evaluation, the cKO group exhibited a lower percentage of freezing time than the WT group, in particular 24 hours and 6 days after the training session, in both sexes. However, the statistical analysis revealed no significant differences due to the *Genotype*, the interaction *Genotype × Time*, or the interaction *Genotype × Sex* (all p > .586).

Nevertheless, there was a main effect of the *Sex* (η_p_^2^ = 0.094; F_1,130_ = 13.39, p < .001), with a higher percentage of freezing in the group of females. There was also a main effect of the *Time* (η_p_^2^ = 0.044; F_3,335_ = 5.97, p < .001), with a statistically significant decrease in the percentage of freezing in the time-points chosen to do the CFC evaluation (p < .001), compared to the freezing displayed by mice after the last footshock.

The interactions *Sex × Time* and *Genotype × Sex × Time* were not significant (all p > .293).

**Fig 4:**
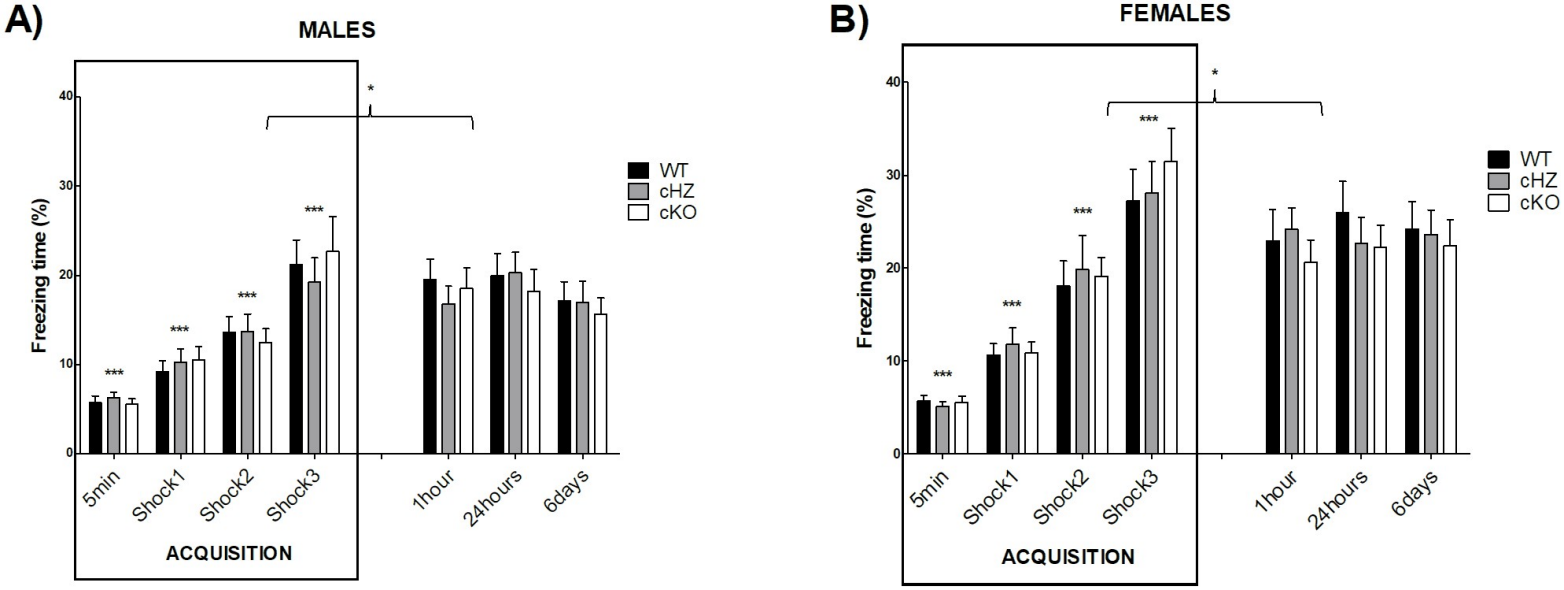
Contextual fear conditioning memory: CFC. The bar plots represent the percentage of freezing (Mean and SEM) during each trial, for the groups WT (*Grik4 Cre+/+*, *SV2A lox/lox*), cHZ (*Grik4 Cre+/-*, *SV2A lox/+*), and cKO (*Grik4 Cre+/-*, *SV2A lox/lox*). Males (n = 22) are presented in subfigure **A**, whereas females (n = 23) are shown in subfigure **B**. Statistically significant differences were found between sex, with p < .001. (***) indicates that each marginal mean (given by the main effect of the factor shock), is significantly different from the rest, being the last one (shock 3) the largest, as yielded by Bonferroni tests taken at p < .001. (*) indicates that the marginal mean corresponding to the shock 3 is significantly different from the marginal means corresponding to the CFC evaluation, as yielded by Bonferroni tests taken at p < .05.

The results of both sessions (acquisition trial and CFC evaluation) are represented in Fig 4.

### Spatial memory: BM

**Fig 5:**
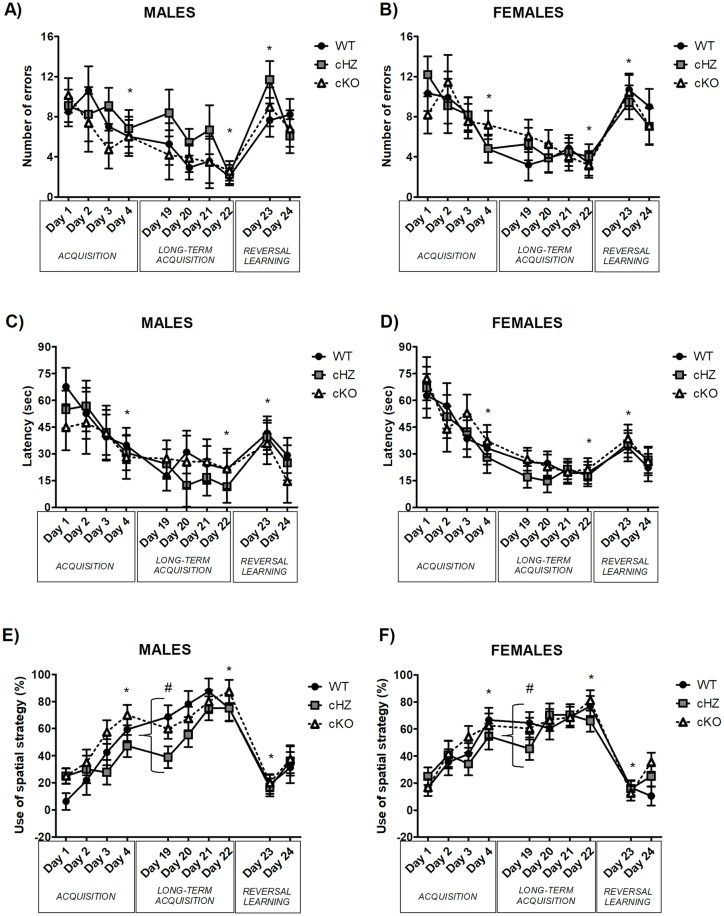
Spatial memory: BM. The lines represent the average per day (4 trials) of the three measured parameters: (1) the number of errors (**A, B**), (2) the latency to find the target hole (**C, D**), and (3) the percentage of use of the spatial strategy (**E, F**) for Males (n = 9) and Females (n = 12). Three groups were compared (Mean and SEM): WT (*Grik4 Cre+/+*, *SV2A lox/lox*), cHZ (*Grik4 Cre+/-*, *SV2A lox/+*), and cKO (*Grik4 Cre+/-*, *SV2A lox/lox*). (*) indicates that the corresponding marginal mean (main effect of *Day*) is significantly different from the previous ones, as yielded by Bonferroni test taken at p < .05. (#) indicates that the corresponding marginal mean (main effect of *Genotype*) is significantly different between the groups WT and cHZ, as yielded by a Tukey HSD test taken at p < .05.

The results of the different trials (acquisition, long-term acquisition and reversal learning) are represented together in Fig 5, to better illustrate the continuous learning of mice.

#### Acquisition trials

There was no significant effect of the variables *Genotype*, *Sex* or the interaction between both in any of the parameters evaluated (number of errors, latency to reach the target hole or percentage of use of the spatial strategy to reach the target), with all p > .538.

However, the analyses highlighted statistically significant differences in all the measures due to the *Days*, with a significant decrease in the number of errors (η_p_^2^ = 0.096; F_2,143_ = 6.16, p = .001) and the latency to reach the target (η_p_^2^ = 0.138; F_2,145_ = 9.26, p < .001), and a significant increase in the percentage of use of the spatial strategy (η_p_^2^ = 0.333; F_2,136_ = 27.41, p < .001).

There was no significant effect of the interaction *Genotype × Days* in the number of errors or in the latency to reach the target (both p > .661), existing a significant difference between the first and the third days in the three genotypes (all p < .049). However, there was a significant effect of the interaction *Genotype × Days* in the percentage of use of the spatial strategy (η_p_^2^ = 0.078; F_5,143_ = 2.32, p = .047). Further Bonferroni’s pairwise comparisons evidenced statistically significant differences between the first and the third days in the WT (p = .004) and in the cKO groups (p > .001), while in the cHZ group these differences did not appeared as significant until the fourth day of acquisition (p = .002).

The interactions *Sex × Days* and *Genotype × Sex × Days* did not appear as significant in any parameter (all p > .441)

#### Probe trial

There were no statistically significant differences due to the variables *Genotype*, *Sex*, or the interaction *Genotype × Sex* neither in the number of errors, the latency or the strategy employed to reach the target (p > .109).

**Fig 6:**
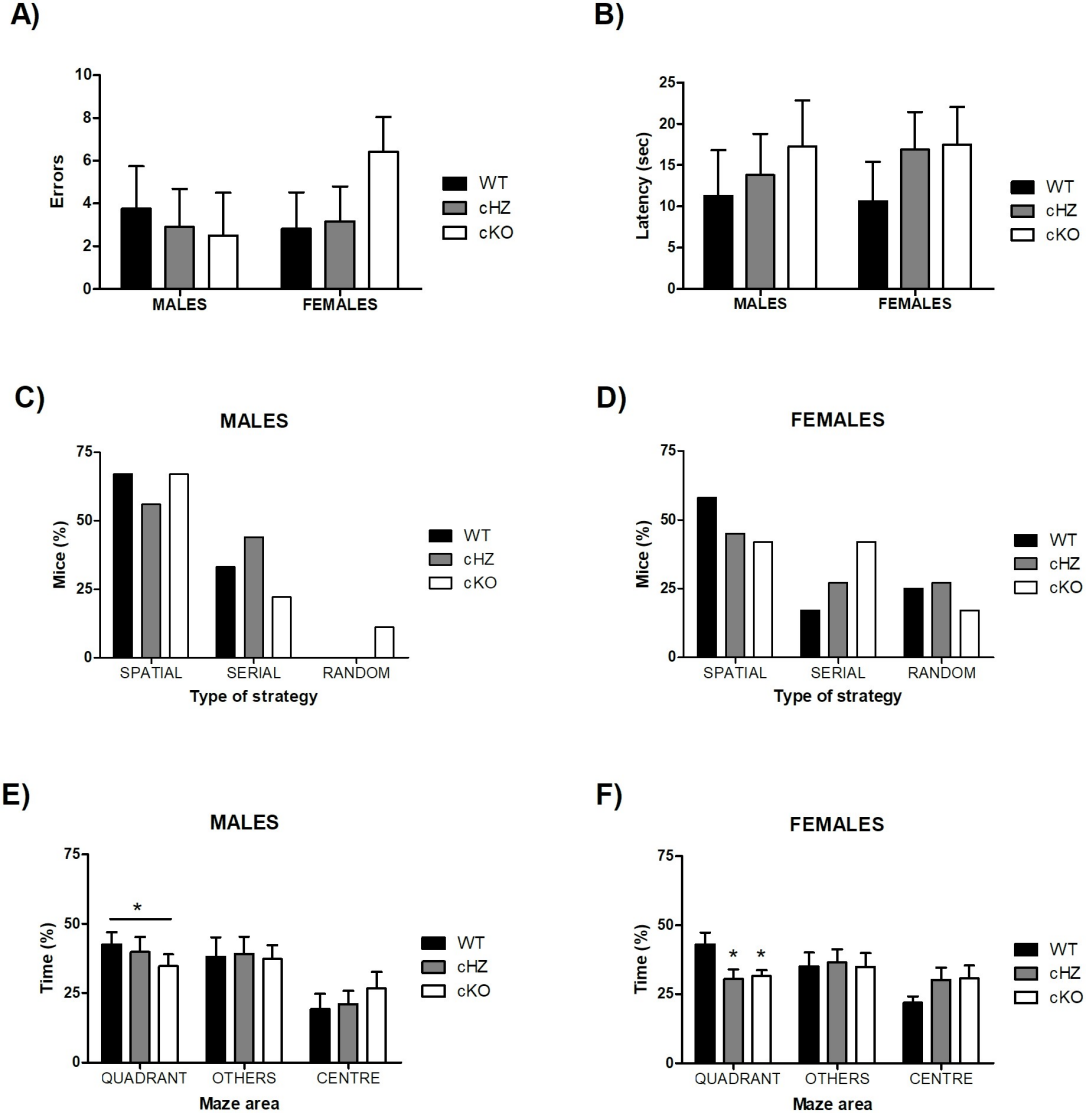
Spatial memory: BM probe. The bar plots (Mean and SEM) represent the number of errors (**A**), the latency to find the target (**B**) and the percentage of mice using the spatial, serial, and random strategies **(C and D)**, and the percentage of time spent in different positions of the maze (quadrant, others, centre) **(E and F)**, for Males (n = 9) and Females (n = 12). Three groups were compared: WT (*Grik4 Cre+/+*, *SV2A lox/lox*), cHZ (*Grik4 Cre+/-*, *SV2A lox/+*), and cKO (*Grik4 Cre+/-*, *SV2A lox/lox*). (*) indicates that the corresponding marginal means of both cKO and cHZ groups are significantly different to the WT group (main effect of *Genotype*), as yielded by a Tukey HSD test taken at p < .05.

However, there was a main effect of the *Genotype* in the percentage of time spend in the quadrant where the target was situated, with η_p_^2^ = 0.108; F_2,55_ = 3.32, p < .043. Further Bonferroni’s pairwise comparison evidenced a difference between the WT and cKO groups, with the latest spending a less percentage of the time during the probe in the quadrant where the target was situated (p = .034) (see Fig 6E and 6F). Although no statistically significant differences were found due to the variable *Sex* or the interaction *Genotype × Sex* (both p > .206), when both sexes were analysed separately the group of females displayed not only significant differences between the WT and cKO groups (p = .030), but also between the WT and cHZ groups (p = .017).

Additionally, there were no statistically significant differences in the percentage of time spent exploring the rest of the holes or in the percentage of time spent in the center of the maze, neither due to the *Genotype*, the *Sex* or the interaction *Genotype × Sex* (all p > .063).

#### Long-term retention

The statistical analysis revealed a significant difference due to the *Genotype* in the percentage of use of the spatial strategy (η_p_^2^ = 0.141; F_2,55_ = 4.50, p = .015), with statistically significant differences between the WT and the cHZ groups (p = .017). This significant effect of the *Genotype* was not present in the number of errors or in the latency to reach the target hole (p < .161). Similarly, there was no significant effect of the *Sex* or the interaction *Genotype × Sex* in any parameter, with p > .380 in all the cases.

#### Long-term acquisition trials

The statistical analysis did not show any significant differences due to the *Genotype*, the *Sex* or the interaction between both variables in any of the parameters evaluated (number of errors, latency to reach the target hole or percentage of use of the spatial strategy to reach the target), with p > .538.

However, there were statistically significant differences due to the *Days* in the number of errors (η_p_^2^ = 0.068; F_2,138_ = 4.24, p = .012), and in the use of the spatial strategy (η_p_^2^ = 0.175; F_3,151_ = 11.46, p < .001). Bonferroni’s pairwise comparison highlighted significant differences in the number of errors between the last two days (p = .030), and in the use of the spatial strategy during the third and the fourth days and the previous ones (both p < .001). There was no effect of the *Days* in the latency to reach the target hole (p = .294).

The interactions *Genotype × Days* and *Sex × Days* and *Genotype × Sex × Days* were not significant in any of the parameters evaluated, with p > .160 for all.

#### Reversal learning

The statistical analysis did not reveal any significant differences due to the *Genotype*, the *Sex* or the interaction *Genotype × Sex* in the number of errors, the latency to reach the target hole, or the percentage of use of the spatial strategy (all p > .084).

However, there were very large statistically significant differences due to the *Days* in the number of errors (η_p_^2^ = 0.519; F_2,110_ = 60.38, p < .001), the latency to reach the target hole (η_p_^2^ = 0.242; F_2,95_ = 16.60, p < .001), and the use of the spatial strategy (η_p_^2^ = 0.693; F_2,96_ = 119.90, p < .001). In the number of errors and the use of the spatial strategy, these differences were present between all the days (all p < .001). Nevertheless, there were no differences in the latency between the last day of long-term acquisition and the last day of reversal learning (p = .365).

The interactions *Genotype × Days*, *Sex × Days* and *Genotype × Sex × Days* were not significant in any of the parameters evaluated, with all p > .196.

## Discussion

During the last few years, the SV2A protein has emerged as a possible key element to understand the epileptic disease. Indeed, when the *SV2A* gene is completely deleted, mice experience seizures starting seven days after birth and die in status epilepticus around day 15 [7,14]. Furthermore, this protein is the molecular target of one of the most prescribed antiepileptic drugs: the levetiracetam [16]. Despite the demonstrated relationship between a decrease in SV2A levels in epileptic foci and the presence of brain seizures, the potential implication of this protein in the cognitive problems exhibited by epileptic patients is barely known. The phenotyping of *Grik4 Cre+/-*, *SV2A lox/lox* (cKO) mice, exempt from spontaneous seizures [30], might therefore help us understand the role of this protein in the cognitive process associated with the hippocampus.

Concerning the spontaneous locomotor activity, our results were similar to those obtained from the heterozygous SV2A mice (+/-) [29], displaying no significant differences attributable to the *Genotype*. Additionally, through the testing procedure, no overt seizure behaviour was observed in the cHZ or the cKO groups, which was consistent with the previously published study regarding these mice [30]. However, cKO and cHZ mice presented a significant different capacity of habituation to a new environment, compared to the WT group. Indeed, we noticed an increased behavioural excitability and anxiety in the transgenic mice, reflected in different adverse reactions to the tests: *SV2A* cKO mice jumped more frequently out of the Barnes maze than WT mice, and they tried to avoid the footshocks using the chamber walls and the ceiling. Moreover, these mice exhibited an increased aggressiveness after being exposed to the contextual fear conditioning test. These emotionally related behaviours have also been reported in chemoconvulsant-induced epileptic models [55].

Due to the link between the hippocampus and the learning and memory [34,56,57], we expected the existence of statistically significant differences between the three groups of mice in the CFC test. Nevertheless, the establishment of a contextual fear conditioning memory was unaffected by the decrease in the SV2A expression. An initial hypothesis is the interference of a memory extinction process, which would disrupt the consolidation and maintenance of the contextual fear conditioning memory. This phenomenon may be caused by a prolonged re-exposure to the context (CS) without receiving the footshocks (US) [58]. However, we did not detect any statistically significant decrease in the percentage of freezing through the CFC evaluation, disproving this hypothesis. Lamberty et al. [29] obtained equivalent results using the passive avoidance procedure: heterozygous SV2A (+/-) mice had similar results than wild type controls at the end of a multi-trial inhibitory avoidance test, even if they have received footshocks along the retention testing. An alternative hypothesis is the existence of compensatory processes, involving the basolateral amygdala complex: even if there is an important role of the hippocampus in the association CS-US [34,56], the amygdala is the main locus in the consolidation and maintenance processes of contextual fear memories [59–61]. In this regard, the cKO mice feature a SV2A decrease only in the hippocampal region, expressing normally this protein in the rest of the brain structures, including the amygdala. The interconnections between the amygdaloid complex and the hippocampus, already described by Pitkänen’s group [62,63], could compensate the SV2A decrease in the hippocampus, explaining the absence of statistically significant differences between groups. A third hypothesis is the possible existence of a compensation phenomenon: in a review published in 2017 concerning the role of the SV2 family members [2], it was proposed that the SV2B expression in glutamatergic neurons could be compensating the SV2A decrease in those neurons, explaining the lack of other statistically significant differences through the testing process.

Despite the absence of differences due to the *Genotype* in the CFC test, sex differences were found in the percentage of freezing time, not only during the acquisition trial, but also during the CFC evaluation. Several studies have explored the underlying causes of these differences, reflecting for example that the dorsal hippocampus is more implicated in males, but there is a preferential recruitment of basal amygdala in the females [64]. Other factors, as the hormones [65], and a different developmental trajectory in the expression of context-mediated freezing [66] also play an important role in the sex differences found in the contextual fear.

On the other hand, the results obtained with the EPM and the BM test highlight statistically significant differences between groups, attributable to the *Genotype*: in the EPM, cKO and cHZ groups entered less in the open arms of the EPM, compared with the WT group. This indicator of anxiety was also found by Lamberty et al. in the heterozygous SV2A mice (+/-) [29]. Indeed, the existence of anxiety and mood disorders has been reported not only in animal models of epilepsy [67,68], but also in patients [69–73]. Additionally, it was found a possible influence of the sex in the anxiety level displayed by the different genotypes (interaction *Sex* × *Genotype*), with significant group differences in males, but not in females. In the literature, the sex-specific role of a protein or a neurotransmitter receptor has barely been studied, with only a few articles describing sex differences in anxiety due to the CB1 and Glu1 receptors [74,75]. Further studies are required to better understand the implication of SV2A in the sexual differences observed in anxiety, since it could have an impact in the choice of the treatment, depending on the sex.

Concerning the BM test, all the genotypes exhibit an equivalent spatial learning through the acquisition trials. However, during the probe test, the cKO group spent less time in the quadrant where the target was located, indicating a possible spatial memory problem associated with the decrease of SV2A in the hippocampal glutamatergic neurons. In females, also the cHZ group exhibit a worse performance compared with the WT group. Furthermore, during the long-term retention trial (day 19), the cHZ group uses the spatial strategy to reach the target significantly less than the WT group, indicating also in this group possible long-term spatial memory problems. In this line, problems in visuospatial memory, olfactory discrimination, and social recognition have also been reported in animal models of epilepsy [55,67,76]. In these models, the memory impairment is commonly evaluated by means of the Morris water maze test [55,76,77]. However, in our case, its use would not be recommended since, in this test, the learning process and the performance are more affected by a high level of anxiety than in the BM [78,79]. Finally, in clinics it has also been described a comorbidity between epilepsy and both mild cognitive impaired [80] and dementia [81].

Additionally, the BM selected procedure allowed us to assess the cognitive flexibility of mice with a two-day reversal learning trials, carried out during the last two days of the procedure. We did not detect statistically significant differences between groups with this complementary analysis, which suggests that the decrease of the SV2A protein in the hippocampus is not interfering in the executive function. However, it is necessary to account for the stress suffered by the animals during the previous tests, which might interfere in the reversal learning evaluation [51]. In conclusion, even if this test can provide us with a first approach to the cognitive flexibility of *SV2A* cKO mice, more accurate analyses with specific behavioural apparatus (e.g. nose-poke portals or a touch-sensitive screen) need to be conducted to specifically assess the executive function [82].

All these results suggest that, in the hippocampal area, the deletion of the *SV2A* gene in one (cHZ mice) or in both alleles (cKO mice) involves statistically significant changes in cognition, including a cognitive impairment and anxiety-related problems, usually present in the epileptic disease [83–89]. However, further studies in epileptic patients should be conducted to confirm the impact of the variations in SV2A protein levels on the severity of the symptomatology.

## Conclusions

The phenotyping of *Grik4 Cre+/-*, *SV2A lox/lox* mice confirms a link between the decrease of the SV2A protein in the hippocampus, and the memory and anxiety-related problems detected in the chronic epilepsy. These results suggest a possible implication of the SV2A protein in anxiety or memory disorders, such as the post-traumatic stress disorder or the Alzheimer’s disease.

## Supporting information

S1 Data EPM Data. Raw data acquired with the EPM test. The columns represent the number and percentage of entries and the time spent (in seconds) in open and closed arms, for the three groups of mice (WT, cHZ, and cKO). On the right, the descriptive statistics for each measured variable.
(XLSX)

S2 Data ACT Data. Raw data acquired with the ACT test. The columns represent the distance travelled (cm) by the three groups (WT, cHZ, and cKO), in each of the three 60-minute session (day1, day2, day3). On the right, the descriptive statistics.
(XLSX)

S3 Data CFC Data. Raw data acquired with the CFC test. The columns represent the percentage of freezing time spent by the three groups (WT, cHZ, and cKO) during the acquisition trial and the CFC evaluation (1h, 24h and 6 days after the acquisition trial). On the right, the descriptive statistics.
(XLSX)

S4 Data BM Data. Raw data acquired with the BM test. The first sheet corresponds to the acquisition trials (days 1–4), the long-term acquisition trials (days 19–22), and the reversal learning trials (RL1 and RL2). The columns represent the number of errors (errors), latency (latence), and the percentage of use of the spatial strategy (perspat) for the three groups of mice (WT, cHZ, and cKO). The second sheet corresponds to the results obtained during the probe trial by the three groups of mice. Four variables have been analysed: errors, latency, time spent in each part of the maze, and strategy. On the right, the descriptive statistics.
(XLSX)

## Abbreviations

η_p_^2^: partial eta squared
ACT: Activity chambers
BM: Barnes Maze
CFC: Contextual Fear Conditioning
cHZ mice: *Grik4 Cre+/-*, *SV2A lox/+*
cKO mice: *Grik4 Cre+/-*, *SV2A lox/lox*
CS: Conditioned stimulus
EPM: Elevated Plus Maze
SV2A: Synaptic Vesicle protein 2A
US: Unconditioned stimulus
WT: *Grik4 Cre+/+*, *SV2A lox/lox*
